# 16S Amplicon Metabarcoding of the Nest Materials of Native Australian Stingless Bees

**DOI:** 10.1128/mra.01181-22

**Published:** 2023-03-13

**Authors:** Boyd Tarlinton, Flavia Carmelina Massaro, Caroline Hauxwell

**Affiliations:** a School of Biology and Environmental Science, Faculty of Science, Queensland University of Technology, Brisbane, Queensland, Australia; Montana State University

## Abstract

We present 16S amplicon data derived from the nest materials of three species of Australian stingless bees (Meliponini). This data set reveals the diversity of bacteria associated with these materials. It will serve as a valuable baseline for further study of the nest microbiome and comparison with the stingless bee microbiota.

## ANNOUNCEMENT

Bacteria associated with nest materials enhance nutrient availability and development of eusocial stingless bees (Meliponini) (1–3). We report the 16S rRNA gene amplicon metabarcoding of nest materials from three Australian meliponines: Austroplebeia australis (Friese), Tetragonula carbonaria (Smith), and Tetragonula hockingsi (Cockerell).

Samples were taken from at least 3 hives of each species at one site in Queensland, Australia (27°21′58.8″S, 152°49′37.0″E): pollen in 2017 and pollen, honey, brood food, and propolis (from brood cell walls) in 2018 and 2019. The hives were resampled each year where possible (Table 1). The materials were harvested aseptically into Eppendorf tubes on ice and stored at −20°C. Brood cells containing 1- to 10-day-old larvae were collected intact and dissected in the lab. Material of each type per hive was pooled and homogenized using aseptic pestles. DNA was extracted using PowerSoil DNA isolation kits (Qiagen, Hilden, Germany).

**TABLE 1:** Metadata and SRA accession numbers for all samples sequenced in this study

Sample ID	Bee species	Hive ID	Material	Date of harvest (day/mo/yr)	Date extracted (day/mo/yr)	Sequencing batch	SRA accession no.
A1-Cerumen-2019-B2	Austroplebeia australis	A1	Propolis	18/12/2019	25/02/2020	B2	SRX18119021
A1-Food-2018-B2	Austroplebeia australis	A1	Food	1/03/2018	25/02/2020	B2	SRX18119011
A1-Honey-2018-B2	Austroplebeia australis	A1	Honey	1/03/2018	25/02/2020	B2	SRX18118998
A1-Pollen-2017-B1	Austroplebeia australis	A1	Pollen	27/02/2017	14/02/2018	B1	SRX18119031
A1-Pollen-2018-B2	Austroplebeia australis	A1	Pollen	1/03/2018	25/02/2020	B2	SRX18118989
A1-Pollen-2019-B2	Austroplebeia australis	A1	Pollen	18/12/2019	25/02/2020	B2	SRX18118982
A2-Cerumen-2019-B2	Austroplebeia australis	A2	Propolis	18/12/2019	25/02/2020	B2	SRX18119022
A2-Food-2018-B2	Austroplebeia australis	A2	Food	1/03/2018	25/02/2020	B2	SRX18119012
A2-Food-2019-B2	Austroplebeia australis	A2	Food	18/12/2019	25/02/2020	B2	SRX18119003
A2-Honey-2018-B2	Austroplebeia australis	A2	Honey	1/03/2018	25/02/2020	B2	SRX18118997
A2-Pollen-2017-B1	Austroplebeia australis	A2	Pollen	27/02/2017	14/02/2018	B1	SRX18119032
A2-Pollen-2018-B2	Austroplebeia australis	A2	Pollen	1/03/2018	25/02/2020	B2	SRX18118988
A2-Pollen-2019-B2	Austroplebeia australis	A2	Pollen	18/12/2019	25/02/2020	B2	SRX18118983
A3-Food-2018-B2	Austroplebeia australis	A3	Food	1/03/2018	25/02/2020	B2	SRX18119013
A3-Honey-2018-B2	Austroplebeia australis	A3	Honey	1/03/2018	25/02/2020	B2	SRX18118996
A3-Pollen-2017-B1	Austroplebeia australis	A3	Pollen	27/02/2017	14/02/2018	B1	SRX18119033
A3-Pollen-2017-B2	Austroplebeia australis	A3	Pollen	27/02/2017	14/02/2018	B2	SRX18119030
A3-Pollen-2018-B2	Austroplebeia australis	A3	Pollen	1/03/2018	25/02/2020	B2	SRX18118987
A4-Food-2019-B2	Austroplebeia australis	A4	Food	18/12/2019	25/02/2020	B2	SRX18119004
A4-Pollen-2019-B2	Austroplebeia australis	A4	Pollen	18/12/2019	25/02/2020	B2	SRX18118994
C1-Cerumen-2019-B2	Tetragonula carbonaria	C1	Propolis	18/12/2019	25/02/2020	B2	SRX18119023
C1-Food-2018-B2	Tetragonula carbonaria	C1	Food	1/03/2018	25/02/2020	B2	SRX18119014
C1-Food-2019-B2	Tetragonula carbonaria	C1	Food	18/12/2019	25/02/2020	B2	SRX18119006
C1-Honey-2018-B2	Tetragonula carbonaria	C1	Honey	1/03/2018	25/02/2020	B2	SRX18119001
C1-Honey-2019-B2	Tetragonula carbonaria	C1	Honey	18/12/2019	25/02/2020	B2	SRX18119043
C1-Pollen-2017-B1	Tetragonula carbonaria	C1	Pollen	27/02/2017	14/02/2018	B1	SRX18119037
C1-Pollen-2018-B2	Tetragonula carbonaria	C1	Pollen	1/03/2018	25/02/2020	B2	SRX18118992
C1-Pollen-2019-B2	Tetragonula carbonaria	C1	Pollen	18/12/2019	25/02/2020	B2	SRX18119005
C2-Cerumen-2019-B2	Tetragonula carbonaria	C2	Propolis	18/12/2019	25/02/2020	B2	SRX18119024
C2-Food-2018-B2	Tetragonula carbonaria	C2	Food	1/03/2018	25/02/2020	B2	SRX18119015
C2-Food-2019-B2	Tetragonula carbonaria	C2	Food	18/12/2019	25/02/2020	B2	SRX18119007
C2-Honey-2018-B2	Tetragonula carbonaria	C2	Honey	1/03/2018	25/02/2020	B2	SRX18119000
C2-Pollen-2017-B1	Tetragonula carbonaria	C2	Pollen	27/02/2017	14/02/2018	B1	SRX18119039
C2-Pollen-2018-B2	Tetragonula carbonaria	C2	Pollen	1/03/2018	25/02/2020	B2	SRX18118991
C2-Pollen-2019-B2	Tetragonula carbonaria	C2	Pollen	18/12/2019	25/02/2020	B2	SRX18119016
C3-Cerumen-2019-B2	Tetragonula carbonaria	C3	Propolis	18/12/2019	25/02/2020	B2	SRX18119025
C3-Food-2018-B2	Tetragonula carbonaria	C3	Food	1/03/2018	25/02/2020	B2	SRX18119017
C3-Honey-2018-B2	Tetragonula carbonaria	C3	Honey	1/03/2018	25/02/2020	B2	SRX18118999
C3-Pollen-2017-B1	Tetragonula carbonaria	C3	Pollen	27/02/2017	14/02/2018	B1	SRX18119040
C3-Pollen-2018-B2	Tetragonula carbonaria	C3	Pollen	1/03/2018	25/02/2020	B2	SRX18118990
C3-Pollen-2019-B2	Tetragonula carbonaria	C3	Pollen	18/12/2019	25/02/2020	B2	SRX18119027
H1-Food-2018-B2	Tetragonula hockingsi	H1	Food	1/03/2018	25/02/2020	B2	SRX18119018
H1-Pollen-2017-B1	Tetragonula hockingsi	H1	Pollen	27/02/2017	14/02/2018	B1	SRX18119034
H1-Pollen-2018-B2	Tetragonula hockingsi	H1	Pollen	1/03/2018	25/02/2020	B2	SRX18118995
H2-Food-2018-B2	Tetragonula hockingsi	H2	Food	1/03/2018	25/02/2020	B2	SRX18119019
H2-Pollen-2017-B1	Tetragonula hockingsi	H2	Pollen	27/02/2017	14/02/2018	B1	SRX18119035
H3-Food-2018-B2	Tetragonula hockingsi	H3	Food	1/03/2018	25/02/2020	B2	SRX18119020
H3-Honey-2018-B2	Tetragonula hockingsi	H3	Honey	1/03/2018	25/02/2020	B2	SRX18119002
H3-Pollen-2017-B1	Tetragonula hockingsi	H3	Pollen	27/02/2017	14/02/2018	B1	SRX18119036
H3-Pollen-2018-B2	Tetragonula hockingsi	H3	Pollen	1/03/2018	25/02/2020	B2	SRX18118993
H4-Cerumen-2019-B2	Tetragonula hockingsi	H4	Propolis	18/12/2019	25/02/2020	B2	SRX18119026
H4-Food-2019-B2	Tetragonula hockingsi	H4	Food	18/12/2019	25/02/2020	B2	SRX18119008
H4-Honey-2019-B2	Tetragonula hockingsi	H4	Honey	18/12/2019	25/02/2020	B2	SRX18118984
H4-Pollen-2019-B2	Tetragonula hockingsi	H4	Pollen	18/12/2019	25/02/2020	B2	SRX18119038
H5-Cerumen-2019-B2	Tetragonula hockingsi	H5	Propolis	18/12/2019	25/02/2020	B2	SRX18119028
H5-Food-2019-B2	Tetragonula hockingsi	H5	Food	18/12/2019	25/02/2020	B2	SRX18119009
H5-Honey-2019-B2	Tetragonula hockingsi	H5	Honey	18/12/2019	25/02/2020	B2	SRX18118985
H5-Pollen-2019-B2	Tetragonula hockingsi	H5	Pollen	18/12/2019	25/02/2020	B2	SRX18119041
H6-Cerumen-2019-B2	Tetragonula hockingsi	H6	Propolis	18/12/2019	25/02/2020	B2	SRX18119029
H6-Food-2019-B2	Tetragonula hockingsi	H6	Food	18/12/2019	25/02/2020	B2	SRX18119010
H6-Honey-2019-B2	Tetragonula hockingsi	H6	Honey	18/12/2019	25/02/2020	B2	SRX18118986
H6-Pollen-2019-B2	Tetragonula hockingsi	H6	Pollen	18/12/2019	25/02/2020	B2	SRX18119042

The 16S rRNA V3 to V4 region was amplified using 341F and 806R primers with Illumina adapters (4), following Applied Biosystems (5) (for the pollen samples in 2017) or Illumina protocols (4) (Table 1). Libraries were assembled using a Nextera XT library preparation kit (Illumina, San Diego, CA) and sequenced on an Illumina MiSeq device with V3 chemistry at CARF (QUT, Brisbane, Australia), generating a total of 14,437,775 pairs of 2 × 300 bp paired-end reads (4).

The demultiplexed sequencing data were processed using QIIME 2 (2022.8) and associated plugins (6). Default parameters were used except where otherwise stated. The DADA2 2022.8.0 plugin (7) was used to trim the paired-end reads, resolve amplicon sequence variants (ASVs), and merge overlapping paired sequences. From the 5′ ends of forward and reverse reads, 17 bp and 21 bp were trimmed, respectively, to remove primer sequences. Reverse reads were truncated 270 bp from the 5′ end due to declining quality scores. Taxonomy was assigned using the BLAST consensus method (8) in the q2-feature-classifier plugin 2022.8.0 (9) using the SILVA 138 SSU 99 NR reference database (10, 11).

Results from QIIME 2 were imported into R 4.2.2 (12) using qiime2R 0.99.6 (13) and used for all downstream analyses. ASVs identified as mitochondria and chloroplasts or unidentified at the kingdom level were removed using phyloseq 1.42.0 (14). Bray-Curtis dissimilarities between samples were calculated from relative ASV abundance using phyloseq. The Bray-Curtis dissimilarity was used for nonmetric multidimensional scaling (NMDS) ordination (k = 4; 1,000 maximum iterations) and PERMANOVA testing (adonis2; sequential terms), both with vegan 2.6-4 (15).

**FIG 1:**
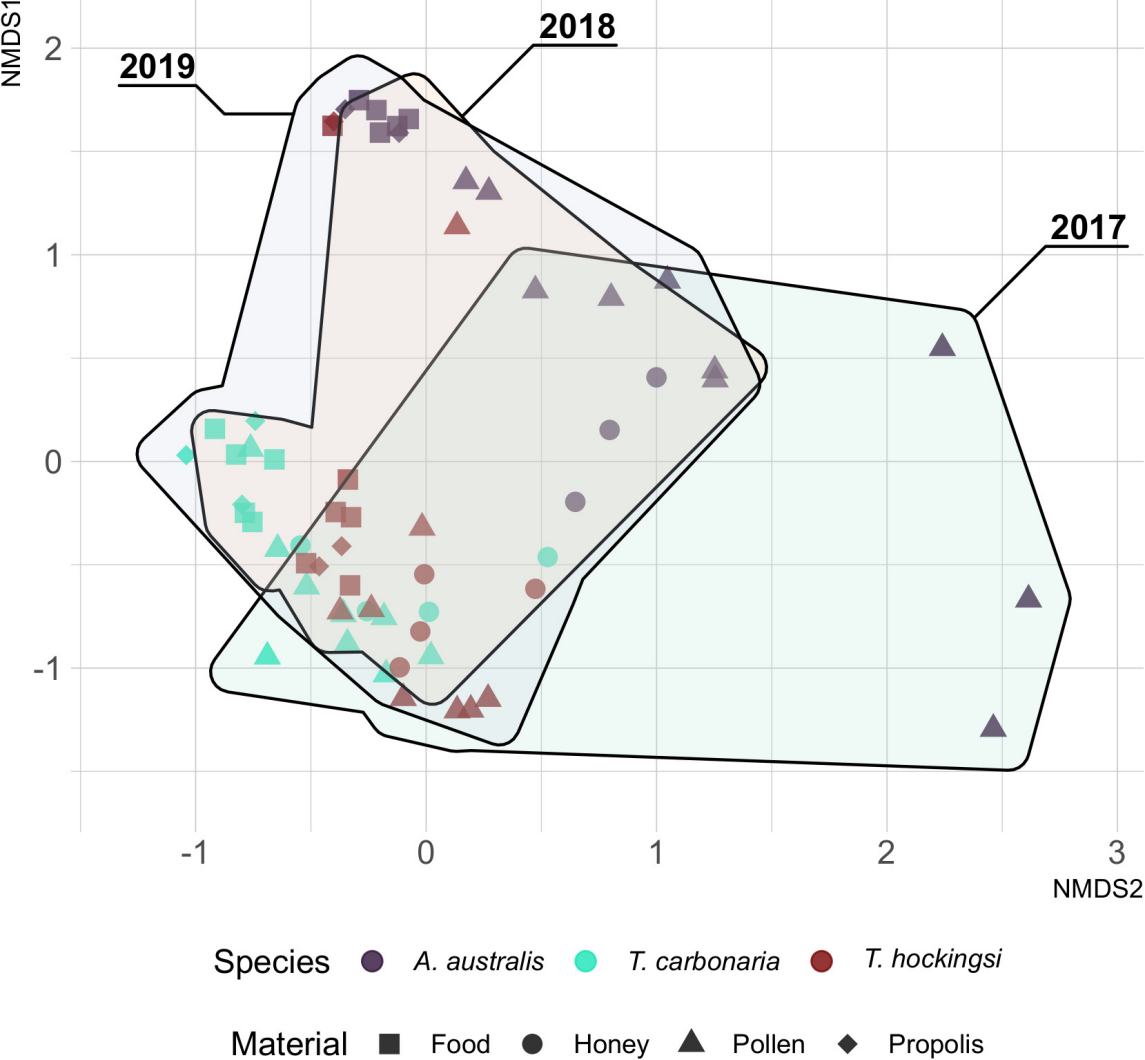
NMDS ordination of samples based on Bray-Curtis dissimilarity (stress, 0.089). Significant determinants of beta diversity (from PERMANOVA): bee species (*R*^2^ = 0.18, *F* = 7.92, *P* = 0.001), material type (*R*^2^ = 0.16, *F* = 4.67, *P* = 0.001), and year (*R*^2^ = 0.04, *F* = 1.95, *P* = 0.004). The individual hive was not significant (*R*^2^ = 0.13, *F* = 1.17, *P* = 0.095). Convex hulls enclose all samples from a given year. Figure drawn using ggplot2 3.4.0 (19), ggforce 0.4.1.9000 (20), and concaveman 1.1.0 (21).

The microbiomes of nest materials of bees from the genus *Tetragonula* clustered separately from those of *A. australis* bees (Fig. 1), mirroring divisions in the bee microbiomes (16). Materials from one *T. hockingsi* hive (H4; 2019) clustered with *A. australis* samples, which might have resulted from environmental transmission of microbes (17) or the usurpation of an *A. australis* hive by a *T. hockingsi* colony (18).

Lactobacilli dominated the nest materials of the *Tetragonula* species, but *Gammaproteobacteria* constituted a significant portion of the *A. australis* honey and pollen microbiome. The bee symbiont *Bombella* spp. (*Acetobacteraceae*) occurred at low abundance in all *A. australis* nest materials, the honey and pollen of both *Tetragonula* species, and *T. hockingsi* propolis (22, 23).

### Data availability.

The samples were uploaded to the NCBI under BioProject accession number PRJNA896876. The accession numbers for all 62 SRA experiments are listed in Table 1. The analysis scripts and intermediate files are available at https://invertebrate-microbiology-group.github.io/Tarlinton_2022_Hive_Microbiome/ and are archived at Zenodo (24).
